# Prostate-Specific Membrane Antigen Expression on PET/CT in Patients with Metastatic Castration-Resistant Prostate Cancer: A Retrospective Observational Study

**DOI:** 10.2967/jnumed.122.264964

**Published:** 2023-06

**Authors:** Letizia Calderoni, Elisa Maietti, Andrea Farolfi, Riccardo Mei, Karly S. Louie, Michael Groaning, Stefano Fanti

**Affiliations:** 1 Nuclear Medicine Division, IRCCS Azienda Ospedaliero-Universitaria di Bologna, Policlinico Di S. Orsola, Bologna, Italy;; 2 Nuclear Medicine, Alma Mater Studiorum, University of Bologna, Bologna, Italy;; 3 Department of Biomedical and Neuromotor Sciences, University of Bologna, Bologna, Italy;; 4 Amgen Ltd., Uxbridge, United Kingdom; and; 5 Amgen Inc., Thousand Oaks, California

**Keywords:** mCRPC, prostate-specific antigen, response to therapy, SUV_max_, [^68^Ga]Ga-PSMA-11 PET/CT

## Abstract

Monitoring therapy response in patients with metastatic castration-resistant prostate cancer (mCRPC) treated with novel hormonal therapies, taxanes, and newly approved therapies is crucial for optimizing treatment. [^68^Ga]Ga-prostate-specific membrane antigen (PSMA)-11 positron emission tomography/computed tomography (PSMA PET/CT) is a promising target for managing treatment in patients with prostate cancer. PSMA is overexpressed in patients with mCRPC; understanding how expression might change in patients undergoing treatment could determine its potential for guiding clinical decisions. We examined PSMA expression in patients with CRPC and compared PET/CT response with prostate-specific antigen (PSA) variation as a prognostic factor for progression-free survival and overall survival (PFS and OS, respectively). **Methods:** This was a single-center, retrospective observational cohort study in patients with CRPC enrolled in the PSMA-PROSTATA registry study (EudraCT: 2015-004589-27). A first and second (if applicable) PSMA PET/CT were performed to determine PSMA expression (absence or presence). PET/CT response was assessed as responders (patients with stable disease, partial or complete response) versus nonresponders (patients with progressive disease) by comparing the first with the second PET/CT. PSA variation (increase or decrease from baseline) was assessed across the same time period. PFS was defined as the time between second PET/CT and PSA recurrence or evidence of radiologic progression. **Results:** Overall, 160 patients with CRPC were included in the analysis. At first PET/CT, nearly all (*n* = 152; 95.0%) patients had PSMA expression (classified as mCRPC), irrespective of prior systemic therapy. SUV_max_ was positively associated with baseline PSA levels and velocity (both *P* < 0.001). According to PET/CT response, median SUV_max_ on first PET/CT was numerically lower in nonresponders than in responders (17.5 vs. 20.4; *P* = 0.127). Similarly, patients with a PSA increase had significantly lower median SUV_max_ on first PET/CT (15.8) than did those with a PSA decrease (30.4; *P* = 0.018). PSA change was, on average, 146% in nonresponders and −57% in responders between first and second PET/CT (*P* < 0.001). Agreement between PET/CT and PSA response was 79% (k = 0.553, *P* < 0.001). Among the 63 patients included in PFS/OS analyses, 76.2% had a relapse and 36.5% died before 24-mo follow-up; median PFS and OS were 6.1 and 24 mo, respectively. PET/CT response, independent of PSA variation, was a significant prognostic factor for PFS. OS was not significantly different between PET/CT responders and nonresponders. **Conclusion:** PSMA PET/CT may be a useful imaging method predictive of treatment response in patients with mCRPC, regardless of ongoing systemic therapy. Data also suggest that response assessed by PET/CT is a potentially more significant prognostic factor than PSA for PFS. Further studies are needed to understand the potential involvement of PSMA expression on survival.

## Visual Abstract

**Figure:**
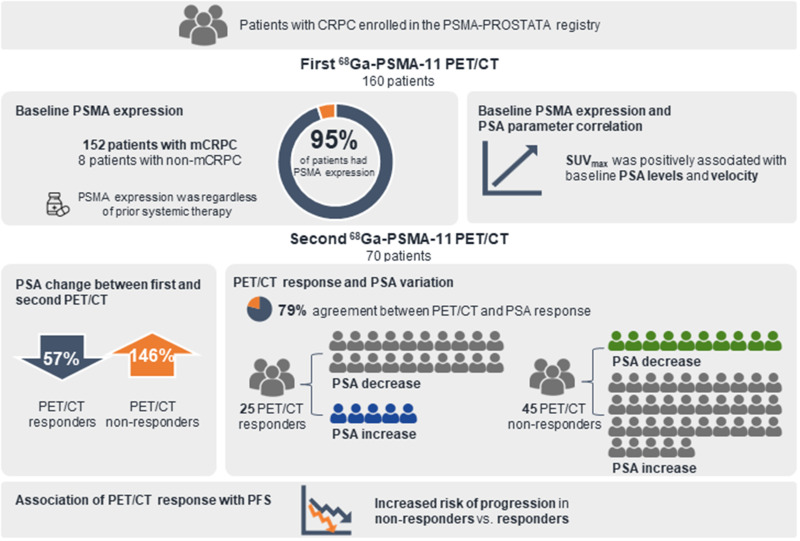

Prostate cancer (PC) is the most commonly occurring cancer in men in Europe (1). Approximately 450,000 new cases were diagnosed in 2018, and the age-standardized mortality rate was 19.4 per 100,000 men. Localized PC may be treated with radiotherapy or surgery; however, many patients develop metastatic disease (2). Initial standard treatment for metastatic PC is androgen deprivation therapy (ADT), with or without chemotherapy (2*,*^3^). Although initially effective, patients gradually become resistant and ultimately progress to metastatic castration-resistant PC (mCRPC), an aggressive disease with a median overall survival (OS) of approximately 2.5 y (2–4). Despite multiple available therapies for mCRPC, the optimal treatment sequence or combinations are unknown (5) and there remains a high unmet need for treatments with novel mechanisms of action.

Radionuclide therapies provide targeted delivery of therapeutic radiation to metastatic PC sites and allow for selection of patients likely to benefit (2). Prostate-specific membrane antigen (PSMA) is overexpressed in most PC cells and levels correlate with disease progression, making it a favorable diagnostic and therapeutic target in mCRPC (2*,*^6^*,*^7^). [^68^Ga]Ga-PSMA-11 positron emission tomography/computed tomography (PSMA PET/CT) can be used to select patients for [^177^Lu]Lu-PSMA-617 therapy, a potential treatment option with demonstrated effectiveness in mCRPC (2*,*^8^*,*^9^).

Monitoring therapy responses in patients with mCRPC treated with novel hormonal therapy, taxanes, and radioligand therapy (RLT) enables clinicians to optimize treatment decisions (5). In PC, there is increasing evidence to support the superiority of PSMA PET/CT over conventional imaging methods and prostate-specific antigen (PSA) serum levels for predicting early response (7*,*^10^*,*^11^). As such, PSMA is emerging as a promising target for PC imaging (12) and might help avoid the administration of costly therapies that are ineffective or not well tolerated.

Currently, data regarding PSMA expression in patients with mCRPC are limited, and it is unclear how treatments may have an impact. The present study aimed to describe PSMA expression in patients with mCRPC and examine whether PET/CT response as compared with PSA variation is a prognostic indicator for progression-free survival (PFS) and OS.

## MATERIALS AND METHODS

### Study Design, Setting, and Participants

This single-center, retrospective observational cohort study was conducted at the Metropolitan Nuclear Medicine Centre of the S. Orsola-Malpighi University Hospital of Bologna, Italy. The study was based on secondary analysis of patients with mCRPC enrolled in the PSMA-PROSTATA registry study (EudraCT: 2015-004589-27) between March 1, 2016, and October 31, 2020, and who underwent [^68^Ga]Ga-PSMA-11 PET/CT between January 2016 and October 2019. Inclusion criteria were: age ≥ 18 y; proven diagnosis of PC; a clinical or biochemical diagnosis of CRPC; and being eligible for second- or subsequent-line therapy. Patients with a history of other tumor diagnosis (i.e., not PC) or with a life expectancy of ≤6 mo (as assessed by each clinician) were excluded.

The Institutional Ethics Committee approved this retrospective study. All participants included in the study were appropriately informed of the purpose of this study and provided signed written informed consent.

### Data Collection and Imaging

Data were collected from medical records at baseline (time of first PET/CT) and during follow-up. Baseline patient characteristics included age, clinical characteristics (Gleason score, pathologic stage, nodal status, tumor burden), treatment history before enrollment in the PSMA-PROSTATA registry, and PSA values (if available within ≤3 mo before baseline). PSA kinetics were calculated using published methodology (13). During follow-up, treatment-related characteristics were collected.

Radiopharmaceutical usage, PET/CT acquisition, and image interpretation were performed as described previously (14). PET images were acquired in accordance with the Joint European Association of Nuclear Medicine and Society of Nuclear Medicine and Molecular Imaging procedure guidelines for PC imaging (15). First and second (if applicable) PET/CT parameters were collected by an experienced physician evaluating the presence of focal uptake suggestive of prostate disease localization, tumor burden, and SUV_max_ of the most significant lesion or lesions. The maximum-intensity-projection and PET/CT fusion images in axial, coronal, and sagittal slices were assessed at the reporting stage.

### Outcomes

The primary outcome was baseline PSMA expression on first PET/CT defined both as SUV_max_ and as the presence of lesions consistent with prostate metastases.

PET/CT response was assessed as responders versus nonresponders by comparing the first with the second PET/CT, as per PSMA PET/CT consensus–based response criteria (16): responders were defined as patients with stable disease, partial response, or complete response; nonresponders were defined as patients with progressive disease. PSA variation (11) between baseline and second PET/CT was assessed as PSA decrease and PSA increase from baseline.

PFS was defined as time to PSA recurrence or evidence of radiologic progression. PFS and OS were calculated starting from the date of the second PET/CT until the date of last visit, death, or end of the study period (i.e., October 31, 2020), whichever occurred first.

### Statistical Analysis

Continuous data were described using median and interquartile range; minimum and maximum values (i.e., range) were also reported in some instances. Categoric data were summarized as absolute and relative frequencies. Statistical significance was considered for a *P* value of less than 0.05.

The overall proportion of patients with PSMA expression on first PET/CT was calculated and reported with binomial 95% CI. SUV_max_ was compared among response and different treatment-related variable groups using the nonparametric Kruskal–Wallis test (>2 groups) or the Wilcoxon–Mann–Whitney test (2 groups); Benjamini and Hochberg correction was applied for multiple comparisons. The relationship between SUV_max_ and PSA parameters (serum level, doubling time, and velocity) was evaluated using Spearman correlation.

PET/CT response was reported for patients who underwent a second PET/CT. Response groups (responders vs. nonresponders) were compared with respect to treatment-related variables, baseline SUV_max_, and PSA level variation from baseline to second evaluation using the Wilcoxon–Mann–Whitney test for continuous data and the χ^2^ test or the Fisher exact test for categoric variables, as appropriate. Concordance between PSA variation and PET/CT response was assessed with Cohen’s κ-coefficient.

In patients who underwent a second PET/CT, PFS and OS analyses were conducted to assess whether PSA variation and PET/CT response were significant predictors. Kaplan–Meier curves were constructed and compared using the log-rank test. A multiple Cox regression model was then estimated to assess whether PSA variation and PET/CT response remained significant after adjustment for age, number of therapy lines, and SUV_max_/PSA baseline value.

Further details on methodology can be found in the supplemental materials (supplemental materials are available at http://jnm.snmjournals.org) (11*,*^13^*,*^16^–^19^).

### Data Sharing

Qualified researchers may request data from Amgen clinical studies. Complete details are available at: https://www.amgen.com/science/clinical-trials/clinical-data-transparency-practices/clinical-trial-data-sharing-request/.

## RESULTS

### Baseline Patient Disposition and Clinical Characteristics

**FIGURE 1.**
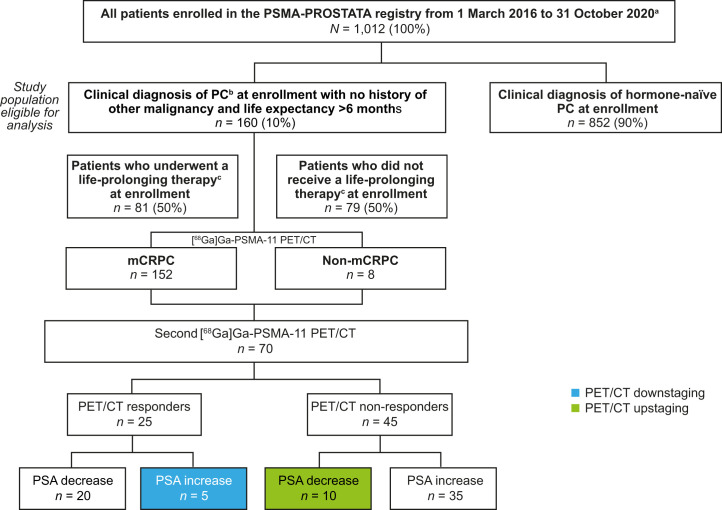
Patient disposition. ^a^PSMA-PROSTATA registry enrollment criteria: patients enrolled in the trial were men with proven diagnosis of PC, who had received radical prostatectomy or radiotherapy as definitive therapy, who had proven biochemical recurrence (defined as 2 consecutive PSA assays ≥ 0.2 ng/mL), who had PSA levels between 0.2 and 2.0 ng/mL, were aged ≥ 35 y, and who had provided written informed consent. Patients were excluded if they were unable to lie flat, to be still, or to tolerate PET/CT scanning or had a history of treatment for another cancer within 1 y before [^68^Ga]Ga-PSMA-11 PET/CT. Use of concomitant therapies, including ADT, were allowed. Follow-up data from routine clinical, pathologic, PET/CT imaging, and PSA levels were collected from patient records. ^b^Patients eligible for study inclusion had proven diagnosis of CRPC as defined by the European Association of Urology guidelines as: serum testosterone < 50 ng/dL or 1.7 nmol/L plus either biochemical progression (rising serum PSA levels) or radiologic progression (appearance of new lesions). ^c^Life-prolonging therapy included novel hormone therapy (abiraterone, enzalutamide), taxanes (docetaxel, cabazitaxel), [^223^Ra]Ra-NaCl, and PSMA-RLT. CRPC = castration-resistant prostate cancer; mCRPC = metastatic castration-resistant prostate cancer; PC = prostate cancer; PET/CT = positron emission tomography/computed tomography; PSA = prostate-specific antigen; PSMA = prostate-specific membrane antigen; PSMA-RLT = α-/β-emitter prostate-specific membrane antigen-radioligand therapy.

A total of 1,012 individuals were enrolled in the PSMA-PROSTATA registry between March 2016 and October 2020. Of these, 160 men with CRPC met the study eligibility criteria and were included in the analysis (Fig. 1). The median age was 72 y (range, 67–77 y), median Gleason score was 8 (range, 7–9), most patients (*n* = 120/160 [75%]) had undergone radical prostatectomy, and 10% had undergone external beam radiation therapy (Table 1). The median time from primary radical treatment to first PET/CT was 6.1 y (range, 2.9–12.2 y). About half of the patients (49.4%; *n =* 79/160) had received ≥1 life-prolonging therapy before enrollment. There was no association between time from radical treatment to baseline PET/CT and number of prior systemic life-prolonging therapies. The median PSA level at first PET/CT was 11.7 ng/mL (interquartile range [IQR], 2–68 ng/mL), median PSA doubling time was 5.2 mo (IQR, 2.9–10.6 mo), and median PSA velocity was 6.8 ng/nL/mo (IQR, 2.5–23.6 ng/nL/mo).

**TABLE 1.** Patient Characteristics

Characteristic	Overall (*N =* 160)	mCRPC (*n =* 152)	nmCRPC (*n =* 8)
Before PET/CT			
Median age (y)	72 (IQR, 67–77)	72 (IQR, 67–76)	77 (IQR, 73–78)
Gleason score (*n*)			
5	3 (1.9%)	3 (2.0%)	0
6	3 (1.9%)	3 (2.0%)	0
7	58 (36.3%)	56 (36.8%)	2 (25.0%)
8	40 (25.0%)	37 (24.3%)	3 (37.5%)
9	53 (33.1%)	50 (32.9%)	3 (37.5%)
10	3 (1.9%)	3 (2.0%)	0
TNM classification (*n*)			
T2	27 (16.9%)	25 (16.4%)	2 (25.0%)
T3	92 (57.5%)	87 (57.2%)	5 (62.5%)
T4	5 (3.1%)	5 (3.3%)	0
Missing	36 (22.5%)	35 (23.0%)	1 (12.5%)
N1	41 (25.6%)	40 (26.3%)	1 (12.5%)
M1	34 (21.3%)	33 (21.7%)	1 (12.5%)
R1	43 (26.9%)	38 (25.0%)	5 (62.5%)
Primary therapy with radical intent (*n*)			
Radical prostatectomy	120 (75.0%)	114 (75.0%)	6 (75.0%)
Associated with PLND	55 (34.4%)	50 (32.9%)	5 (62.5%)
EBRT	16 (10.0%)	15 (9.9%)	1 (12.5%)
Adjuvant/salvage radiotherapy (*n*)	88 (55.0%)	85 (55.9%)	3 (37.5%)
Median time from primary radical treatment to PET/CT (y)	6.1 (IQR, 2.9–12.2)	6.1 (IQR, 2.8–12.6)	7.5 (IQR, 4.9–10.9)
No. of prior systemic life-prolonging therapies (*n*)*			
No systemic therapy	81 (50.6%)	74 (48.7%)	7 (87.5%)
1 therapy line	38 (23.8%)	37 (24.3%)	1 (12.5%)
2 therapy lines	22 (13.8%)	22 (14.5%)	0
≥3 therapy lines	19 (11.9%)	19 (12.5%)	0
At time of first PET/CT			
Median PSA (ng/mL)	11.7 (IQR, 2–68)	13.1 (IQR, 2.1–77)	1.0 (IQR, 0.5–4.1)
Median PSA doubling time (mo)	5.2 (IQR, 2.9–10.6)	5.0 (IQR, 2.9–10.6)	6.4 (IQR, 5.9–12.3)
>6 mo (*n*)	71 (44.4%)	66 (43.4%)	5 (62.5%)
>10 mo (*n*)	43 (26.9%)	41 (27.0%)	2 (25.0%)
Median PSA velocity (ng/nL/mo)	6.8 (IQR, 2.5–23.6)	7.8 (IQR, 2.7–25.5)	1.5 (IQR, 0.5–4.5)
Ongoing second-line therapy (*n*)	19 (11.9%)	19 (12.5%)	0
Ongoing ADT (*n*)^†^	106 (66.3%)	101 (66.4%)	5 (62.5%)

* Excluding ADT.

† At time of PSMA ongoing ADT is < 90 d.

ADT = androgen deprivation therapy; EBRT = external beam radiation therapy; IQR = interquartile range; PET/CT = positron emission tomography/computed tomography; PSA = prostate-specific antigen; nm/mCRPC = nonmetastatic/metastatic castration-resistant prostate cancer; PLND = pelvic lymph node dissection; TNM = tumor, node, metastasis.

### Baseline PSMA Expression

PSMA expression at first PET/CT was confirmed in 152 of 160 (95.0%) patients (95% CI, 90.4–97.8); these patients were classified as having mCRPC. The distribution of metastatic sites in patients with mCRPC is shown in Supplemental Table 1. PSMA expression at first PET/CT varied by site of relapse or metastasis (Table 2); SUV_max_ was significantly higher in metastasis involving bone than in relapse or metastasis of other sites (*P*-adjusted = 0.023 vs. nodes; *P*-adjusted = 0.003 vs. prostate bed relapse; *P*-adjusted = 0.047 vs. visceral). SUV_max_ was significantly lower in patients with prostate bed relapse than in those with node lesions (*P*-adjusted = 0.023).

**TABLE 2.** PSMA Expression at First PET/CT by Site of Relapse/Metastasis (*n =* 152)

Site of relapse/metastasis	No. of patients (*n*)	Median SUV_max_	Min–max SUV_max_
Bones	100 (65.8%)	24.0 (IQR, 14.3–37.9)	3.8–127.0
Nodes*	91 (59.9%)	16.7 (IQR, 11.0–28.0)	2.1–138.0
Prostate bed relapse	32 (21.1%)	10.1 (IQR, 7.9–19.9)	3.0–68.0
Visceral	22 (14.5%)	17.5 (IQR, 6.0–27.0)	4.6–45.0

* Pelvic and distant.

IQR = interquartile range; PET/CT = positron emission tomography/computed tomography; PSMA = prostate-specific membrane antigen; SUV_max_ = maximized standardized uptake value.

Of the 79 of 160 patients who received systemic therapy before their first PET/CT, 78 (98.7%) expressed PSMA; among the 81 patients who did not receive prior systemic therapy (excluding ADT), 74 (91.4%) expressed PSMA (between-group difference *P* = 0.075). Baseline SUV_max_ was significantly higher in patients who received systemic treatment before first PET/CT than in those who did not (*P* = 0.009; Table 3). There was no significant difference in SUV_max_ by type of last systemic treatment received before first PET/CT.

**TABLE 3.** PSMA Expression at First PET/CT by Systemic Treatment (*n =* 160)

Variable	No. of patients (*n*)	Proportion of patients expressing PSMA* (*n*)	Median SUV_max_ in lesion with highest value	*P*
Received systemic treatment before first PET/CT^†^				0.009
No	81 (50.6%)	74 (91.4%)	16.7 (IQR, 8.0–34.0)	
Yes	79 (49.4%)	78 (98.7%)	29.0 (IQR, 15.0–37.7)	
No. of prior therapy lines if received systemic treatment before first PET/CT				0.087
1	38 (23.8%)	37 (97.4%)	19.9 (IQR, 12.9–37.0)	
2	22 (13.8%)	22 (100%)	32.4 (IQR, 20.4–37.0)	
3	13 (8.1%)	13 (100%)	29.0 (IQR, 20.0–39.0)	
4	6 (3.8%)	6 (100%)	25.9 (IQR, 9.8–38.0)	
Last systemic treatment before first PET/CT				0.698
Abiraterone or enzalutamide	28 (17.5%)	28 (100%)	24.2 (IQR, 13.9–39.0)	
Docetaxel or cabazitaxel	24 (15.0%)	23 (95.8%)	27.5 (IQR, 15.3–37.4)	
Palliative, [^223^Ra]Ra-NaCl or PSMA-RLT	27 (16.9%)	27 (100%)	30.0 (IQR, 17.0–39.0)	
Ongoing systemic treatment at time of first PET/CT				0.931
No	141 (88.1%)	133 (94.3%)	20.4 (IQR, 12.0–37.0)	
Yes	19 (11.9%)	19 (100%)	19.7 (IQR, 9.9–35.0)	
Second PET/CT				0.277
No	90 (56.3%)	86 (95.6%)	26 (IQR, 14–37)	
Yes	70 (43.8%)	66 (94.3%)	18 (IQR, 9.2–37.7)	

* SUV_max_ > 2.1.

† ADT was not considered.

IQR = interquartile range; PET/CT = positron emission tomography/computed tomography; PSMA = prostate-specific membrane antigen; PSMA-RLT = α-/β-emitter prostate-specific membrane antigen-radioligand therapy; SUV_max_ = maximized standardized uptake value.

### Correlation of Baseline PSMA Expression and PSA Parameters

SUV_max_ at first PET/CT was significantly and positively associated with baseline serum PSA levels (Spearman ρ, 0.377; *P* < 0.001) and PSA velocity (Spearman ρ, 0.294, *P* < 0.001), but not with PSA doubling time (Spearman ρ, −0.071; *P* = 0.373). When analyzed according to last systemic treatment received before first PET/CT, positive associations were observed for SUV_max_ at first PET/CT and baseline serum PSA levels in subgroups who received abiraterone/enzalutamide or no systemic treatment (*P* = 0.011 and *P* < 0.001, respectively). There was no association in subgroups who received docetaxel/cabazitaxel or palliative/[^223^Ra]Ra-NaCl/PSMA-RLT (α-/β-emitter prostate-specific membrane antigen–radioligand therapy).

### Baseline PSMA Expression According to PET/CT Response and PSA Variation

Overall, 70 patients underwent a second PET/CT scan: 45 patients (64.3%) were nonresponders and 25 (35.7%) were responders (Supplemental Table 2). There was no significant difference in median time from first to second PET/CT scans between nonresponders and responders (8.5 [IQR, 6.4–12.2] vs. 5.8 [IQR, 5.0–13] mo, respectively; *P* = 0.216).

Nonresponders had numerically lower median SUV_max_ on first PET/CT than responders (17.5 [IQR, 12.0–55.5] vs. 20.4 [IQR, 8.0–35.0], respectively; *P* = 0.127) (Supplemental Table 3). Similarly, nonresponders had numerically lower median SUV_max_ on first PET/CT than responders in those who received the same therapy before and after first PET/CT (*P* = 0.064). In patients who received different therapy before and after first PET/CT, there was no significant difference in median SUV_max_ between nonresponders and responders (*P* = 0.568). Considering the last treatment type before second PET/CT, there were no significant differences in SUV_max_ between nonresponders and responders in any of the treatment subgroups (abiraterone/enzalutamide, docetaxel/cabazitaxel/chemotherapy or palliative/[^223^Ra]Ra-NaCl/PSMA-RLT).

Patients with a PSA decrease between first and second PET/CT had significantly higher median SUV_max_ on first PET/CT versus patients with a PSA increase (30.4 [IQR, 13.0–55.5] vs. 15.8 [IQR, 7.3–27.6], respectively; *P* = 0.018; Supplemental Table 3). This was particularly evident in patients who received different treatment before and after first PET/CT (*P* = 0.039). Differences were also observed in the subgroup who received docetaxel/cabazitaxel/chemotherapy before second PET/CT (*n =* 15, *P* = 0.068).

There was a significant difference in PSA change between nonresponders and responders at second PET/CT (*P* < 0.001). The median change in PSA between first and second PET/CT was 146% (IQR, 15.6–463) in nonresponders and −56.9% (IQR, −4.6 to −16.6) in responders. Analysis of concordance showed a 78.6% agreement between PET/CT response and PSA variation, significantly higher than expected from random chance (Cohen’s k = 0.553, *P* < 0.001; Supplemental Fig. 1). However, 5 of 70 patients (7.1%) were responders according to second PET/CT but reported an increase in PSA, and 10 of 70 (14.3%) were nonresponders according to second PET/CT but reported a decrease in PSA.

### Association of PET/CT Response and PSA Variation with PFS and OS

Sixty-three patients with follow-up after second PET/CT were included in analyses of PFS and OS. Of these, 48 (76.2%) patients had a relapse, 23 (36.5%) died, and 33 (52.4%) were lost before 24-mo follow-up. The median PFS was 6.1 mo, and 24-mo OS was 49%.

**FIGURE 2.**
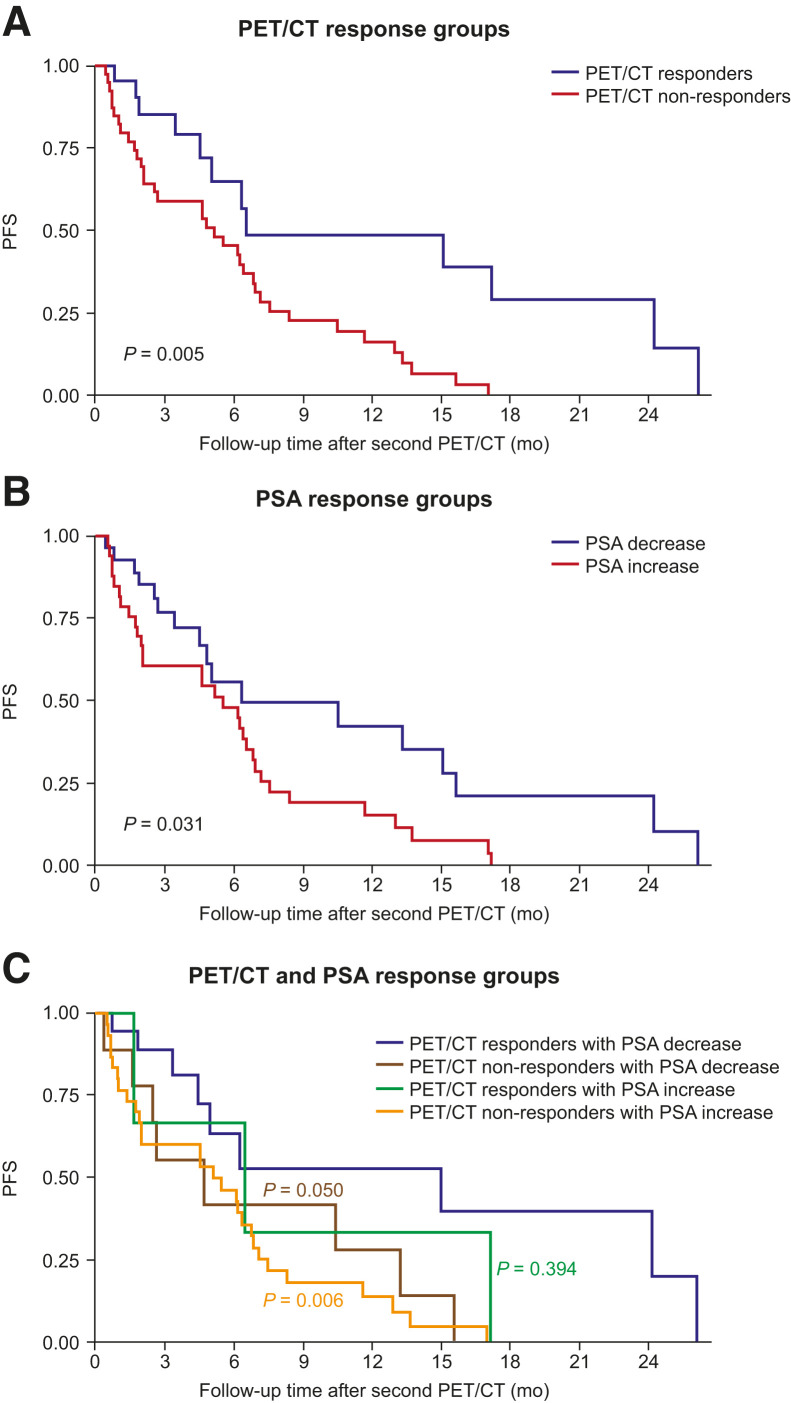
Association of PFS after second PET/CT with PET/CT response (A), change from baseline in serum PSA level (B), and combination of PET/CT response and change from baseline in serum PSA level (C) (*n =* 63). mo = months; PET/CT = positron emission tomography/computed tomography; PFS = progression-free survival; PSA = prostate-specific antigen.

PFS was significantly different between PET/CT response groups (log-rank test *P* = 0.005; Fig. 2A). After adjusting for SUV_max_ at first PET/CT, age, and number of therapy lines, PET/CT nonresponders showed an increased risk of progression compared with responders (hazard ratio [HR], 3.0 [95% CI, 1.4–6.7]; *P* = 0.006). PFS was also significantly different between PSA variation groups (i.e., increase vs. decrease from baseline; log-rank test *P* = 0.031; Fig. 2B). After adjusting for PSA at baseline, age, and number of therapy lines, patients with a PSA increase between first and second PET/CT had a higher risk of progression than did those with a decrease (HR, 2.1 [95% CI, 1.0–4.7]; *P* = 0.059).

**FIGURE 3.**
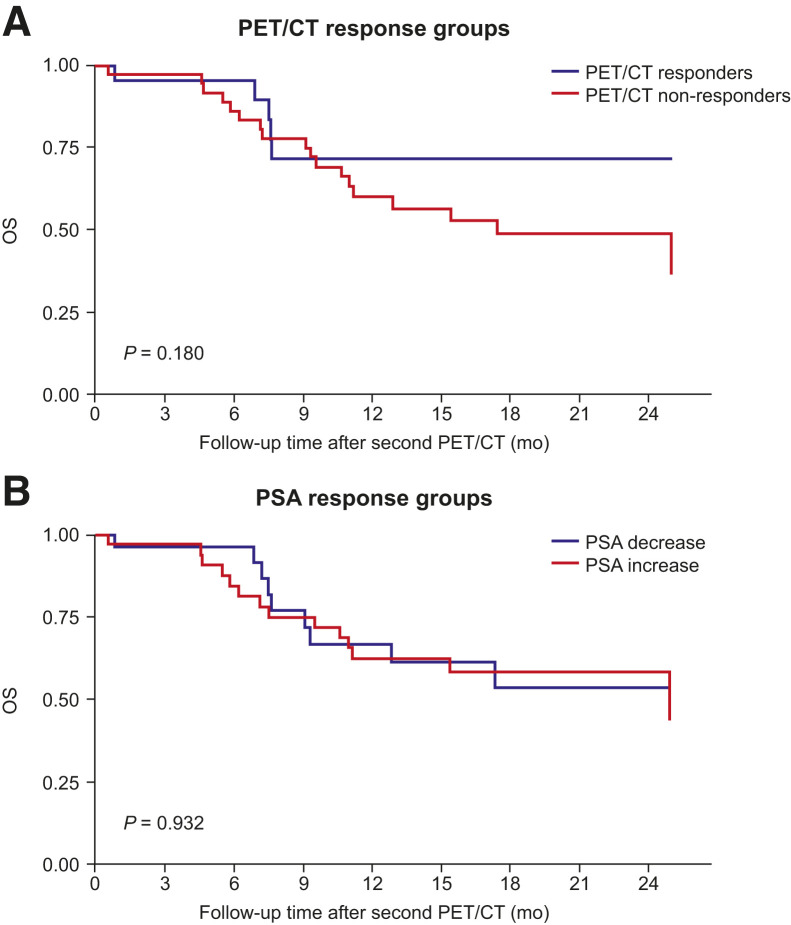
Association of OS with PET/CT response (A) and change from baseline in serum PSA level (B) (*n =* 63). mo = months; OS = overall survival; PET/CT = positron emission tomography/computed tomography; PSA = prostate-specific antigen.

When PET/CT response and PSA variation groups were combined, PET/CT nonresponders with an increase in PSA had a significantly higher risk of disease progression than did PET/CT responders with a decrease in PSA (HR, 3.4 [95% CI, 1.4–8.0]; *P* = 0.006; Fig. 2C). PET/CT nonresponders with a decrease in PSA were also at higher risk of progression than were PET/CT responders with a decrease in PSA (HR, 2.8 [95% CI, 1.0–8.0]; *P* = 0.050). There was no difference in progression risk in PET/CT responders with an increase in PSA compared with PET/CT responders with a decrease in PSA (HR, 1.8 [95% CI, 0.5–7.0]; *P* = 0.394). PET/CT nonresponders appeared to have lower OS than responders (Fig. 3A); however, the difference between the 2 Kaplan–Meier curves was not significant (*P* = 0.180). There was no difference in OS between patients with a decrease in PSA and those with an increase (*P* = 0.932; Figure 3B).

## DISCUSSION

The present retrospective analysis of a large, single-center registry examined PSMA expression in patients with CRPC. Monitoring therapy response is important for treatment decisions in patients with mCRPC, and previous evidence suggest that PET/CT may aid in predicting early response to therapy (7*,*^10^*,*^11^*,*^20^*,*^21^); however, data are limited. Findings in this study suggest that PSMA expression on PET/CT could be more prognostic than PSA parameters for PFS and might be a promising tool for guiding clinical decisions in patients with advanced PC.

Consistent with the literature, most patients (95%) in our analysis had PSMA expression at baseline, as determined by PET/CT imaging (6). Interestingly, PSMA expression was higher in patients with prior systemic treatment, although this was irrespective of the number and type of therapy line. Higher PSMA expression in these patients may be due to more advanced and aggressive disease. The type of prior treatment did not influence PSMA expression; of particular note, there was no difference in PSMA expression in the 18% of patients last treated with abiraterone or enzalutamide before first PET/CT compared with patients last treated with other systemic therapies. Studies have shown that enzalutamide may affect the expression of PSMA on the PC cell surface early after treatment initiation (from 14 to 25 d) (22*,*^23^). Conversely, PSMA variations in patients treated with abiraterone or enzalutamide were not observed when treated over a longer period (87–110 d) (24), suggesting that the upregulation of PSMA expression after abiraterone or enzalutamide is transient. These findings are in line with the absent effect of abiraterone or enzalutamide on PSMA expression in our study, although further research is needed given the small number of patients. Variations in PSMA expression were observed according to the site of relapse or metastasis, and the highest SUV_max_ occurred in metastasis involving the bone. It should be noted that the study included both patients who did and patients who did not undergo radical prostatectomy, which may explain why a relatively high proportion of patients had prostate bed relapse (21%).

As expected, we found that PSMA expression at first PET/CT correlated with PSA levels and velocity, but not doubling time. We also confirmed the general relationship understood to exist between high PSMA expression and advanced stage disease (6).

However, higher PSMA expression may also correlate with treatment response; in a retrospective study conducted in patients with mCRPC who had received [^177^Lu]Lu-PSMA-617, higher PSMA expression was associated with longer OS, longer PFS, and higher PSA variation (25). In our analysis, PET/CT nonresponders had numerically lower SUV_max_ on first PET/CT than responders. The lack of statistical significance may relate to the low number of patients who underwent a second PET/CT. There was also no significant difference in SUV_max_ between nonresponders and responders when analyzed by last treatment received before second PET/CT. Patients with an increase in PSA levels had significantly lower SUV_max_ at baseline than patients with a PSA decrease, with good agreement between PET/CT response and PSA variation. These findings are consistent with another retrospective study that demonstrated correlations between SUV_max_ and PSA response in patients with mCRPC (21), suggesting that PSMA expression on PET/CT may be a predictive marker of treatment response. This could potentially enable better patient selection for therapies targeting PSMA; patients with lower expression at baseline are less likely to respond to further lines of therapy, possibly due to more aggressive and undifferentiated disease.

Previous studies have demonstrated a higher accuracy of PET/CT in patients with CRPC compared with biochemical response and other conventional methods, supporting its utility as a reliable parameter to predict response to systemic treatment for mCRPC (7*,*^10^*,*^11^*,*^20^*,*^21^). Although 1 study reported that the performance of PET/CT was not superior to conventional imaging in differentiating progressive disease from response to treatment, this may be due to the small number of patients involved (20). Our analysis suggests that PET/CT might be more reliable than PSA for predicting response to therapy; however, our findings were not statistically significant given the small sample size.

Response at first PET/CT and PSA decrease from baseline were both significant prognostic factors for PFS. The combined analysis suggested that PET/CT response may be a more significant prognostic factor than PSA variation. In line with recommendations from the Prostate Cancer Clinical Trials Working Group 3 (26), this suggests that therapy should not be discontinued based only on PSA variation. As PSA may not always predict response to therapy, PET/CT may be a more reliable option for early prediction; however, the burden of disease or response to therapy may be underestimated if the timing of PET/CT is not optimal (27). Consensus is needed on the appropriate point at which to repeat PET/CT.

In the current analysis, we did not observe any relation between PET/CT response or PSA variation and OS in patients with mCRPC. A retrospective study in patients with mCRPC reported similar results, with no correlations observed between PET parameters and OS (21); however, the findings may be explained by the limited number of patients included in these analyses.

As with all retrospective single-centered studies, our findings may not be representative of the general population with mCRPC. The retrospective design and consequent number of patients lost to follow-up also mean that associations between PET/CT response and OS should be interpreted with caution. Further, assessment of response to therapy was not possible in patients without a second PET/CT. A further limitation is the heterogeneity of the cohort of enrolled patients, in terms of therapy management, baseline characteristics, and enrollment. Only those with a suspicion of progression were included, and consequently, a substantial proportion of patients did not undergo PSMA PET/CT during the study period; however, the use of established imaging protocols implemented by experienced operators is a strength of the study.

Finally, in advanced PC, pre- and postdiagnosis management can vary as there are no precise guidelines on the order and duration of second-line therapies, and serious adverse effects may be experienced, particularly by elderly patients and those with comorbidities. This complicates the interpretation of data on the efficacy and usefulness of diagnostic investigations. The ability of PSMA PET/CT to detect recurrence at an earlier stage of disease suggests greater opportunities for life-prolonging treatment; however, given the often indolent clinical course of recurrent PC, the potential benefits of earlier, aggressive therapeutic intervention in patients with limited recurrence will need to be weighed carefully against the risk of associated toxicities and quality of life impairment (28).

## CONCLUSION

Our findings suggest that PSMA expression on PET/CT may be a predictive marker of treatment response in patients with mCRPC regardless of ongoing systemic therapy at the time of PET/CT. The data also suggest that PET/CT response is a more significant prognostic factor for PFS than PSA variation; however, larger studies are warranted to confirm these findings and to further explore PSMA expression in relation to patient survival.

## DISCLOSURE

This study was funded by Amgen Inc. (Thousand Oaks, California, USA), which paid the fee to publish the article’s open access and played a role in the study design, data collection and analysis, decision to publish, and preparation of the manuscript. Karly S. Louie was employed by Amgen Ltd. at the time the study was conducted, holds stocks in Amgen, and is an employee of and holds stock options/shares in BioMarin Pharmaceutical Inc. Michael Groaning is an employee of and holds stock in Amgen. Stefano Fanti has consulted for AAA, Amgen, Astellas, AstraZeneca, Bayer, GE, Janssen, Novartis, Sofie, and Telix and has received research funding and travel support from AAA, Amgen, Astellas, AstraZeneca, Bayer, GE, Janssen, Novartis, Sofie, and Telix. Funding for medical writing support for this article was provided by Amgen Ltd (Uxbridge, UK). No other potential conflict of interest relevant to this article was reported.

## Notes

KEY POINTS **QUESTION:** What is the prognostic value (for OS and PFS) of PSMA PET response and how does this compare with PSA response? **PERTINENT FINDINGS:** In this retrospective, observational study, nearly all patients with CRPC (95%) had PSMA expression on PET/CT, regardless of prior systemic therapy, and were classified as having mCRPC. PSMA expression was significantly associated with PSA level and velocity; PSA change between first and second PET/CT was 146% in nonresponders and −57% in responders, with a 79% agreement between PET/CT and PSA response; and PET/CT response, despite PSA variation, was a significant prognostic factor for PFS. **IMPLICATIONS FOR PATIENT CARE:** Understanding the impact of treatment on PSMA expression could inform utility of [^68^Ga]Ga-PSMA-11 PET/CT for guiding clinical decisions in patients with mCRPC.

## References

[1] Ferlay J Colombet M Soerjomataram I. Cancer incidence and mortality patterns in Europe: estimates for 40 countries and 25 major cancers in 2018. Eur J Cancer. 2018;103:356–387. 30100160 10.1016/j.ejca.2018.07.005

[2] Hofman MS Emmett L Violet J. TheraP: a randomized phase 2 trial of (177) Lu-PSMA-617 theranostic treatment vs cabazitaxel in progressive metastatic castration-resistant prostate cancer (Clinical Trial Protocol ANZUP 1603). BJU Int. 2019;124(suppl 1):5–13. 31638341 10.1111/bju.14876

[3] Galletti G Leach BI Lam L Tagawa ST. Mechanisms of resistance to systemic therapy in metastatic castration-resistant prostate cancer. Cancer Treat Rev. 2017;57:16–27. 28527407 10.1016/j.ctrv.2017.04.008

[4] Claps M Mennitto A Guadalupi V. Immune-checkpoint inhibitors and metastatic prostate cancer therapy: learning by making mistakes. Cancer Treat Rev. 2020;88:102057. 32574991 10.1016/j.ctrv.2020.102057

[5] Parker C Castro E Fizazi K. Prostate cancer: ESMO clinical practice guidelines for diagnosis, treatment and follow-up. Ann Oncol. 2020;31:1119–1134. 32593798 10.1016/j.annonc.2020.06.011

[6] Ruigrok EAM van Weerden WM Nonnekens J de Jong M. The future of PSMA-targeted radionuclide therapy: an overview of recent preclinical research. Pharmaceutics. 2019;11:560. 31671763 10.3390/pharmaceutics11110560 PMC6921028

[7] Hofman MS Lawrentschuk N Francis RJ. Prostate-specific membrane antigen PET-CT in patients with high-risk prostate cancer before curative-intent surgery or radiotherapy (proPSMA): a prospective, randomised, multicentre study. Lancet. 2020;395:1208–1216. 32209449 10.1016/S0140-6736(20)30314-7

[8] Hofman MS Emmett L Sandhu S. [^177^Lu]Lu-PSMA-617 versus cabazitaxel in patients with metastatic castration-resistant prostate cancer (TheraP): a randomised, open-label, phase 2 trial. Lancet. 2021;397:797–804. 33581798 10.1016/S0140-6736(21)00237-3

[9] Hofman MS Violet J Hicks RJ. [^177^Lu]-PSMA-617 radionuclide treatment in patients with metastatic castration-resistant prostate cancer (LuPSMA trial): a single-centre, single-arm, phase 2 study. Lancet Oncol. 2018;19:825–833. 29752180 10.1016/S1470-2045(18)30198-0

[10] Lawal IO Mokoala KMG Mahapane J. A prospective intra-individual comparison of [^68^Ga]Ga-PSMA-11 PET/CT, [^68^Ga]Ga-NODAGA(ZOL) PET/CT, and [^99m^Tc]Tc-MDP bone scintigraphy for radionuclide imaging of prostate cancer skeletal metastases. Eur J Nucl Med Mol Imaging. 2021;48:134–142. 32424485 10.1007/s00259-020-04867-y

[11] Grubmüller B Senn D Kramer G. Response assessment using ^68^Ga-PSMA ligand PET in patients undergoing ^177^Lu-PSMA radioligand therapy for metastatic castration-resistant prostate cancer. Eur J Nucl Med Mol Imaging. 2019;46:1063–1072. 30569186 10.1007/s00259-018-4236-4 PMC6451716

[12] Farolfi A Koschel S Murphy DG Fanti S. PET imaging in urology: a rapidly growing successful collaboration. Curr Opin Urol. 2020;30:623–627. 32701721 10.1097/MOU.0000000000000800

[13] Khan MA Carter HB Epstein JI. Can prostate specific antigen derivatives and pathological parameters predict significant change in expectant management criteria for prostate cancer? J Urol. 2003;170:2274–2278. 14634395 10.1097/01.ju.0000097124.21878.6b

[14] Farolfi A Ceci F Castellucci P. ^68^Ga-PSMA-11 PET/CT in prostate cancer patients with biochemical recurrence after radical prostatectomy and PSA <0.5 ng/mL. Efficacy and impact on treatment strategy. Eur J Nucl Med Mol Imaging. 2019;46:11–19. 29905907 10.1007/s00259-018-4066-4

[15] Fendler WP Eiber M Beheshti M. ^68^Ga-PSMA PET/CT: Joint EANM and SNMMI procedure guideline for prostate cancer imaging: version 1.0. Eur J Nucl Med Mol Imaging. 2017;44:1014–1024. 28283702 10.1007/s00259-017-3670-z

[16] Fanti S Goffin K Hadaschik BA. Consensus statements on PSMA PET/CT response assessment criteria in prostate cancer. Eur J Nucl Med Mol Imaging. 2021;48:469–476. 32617640 10.1007/s00259-020-04934-4 PMC7835167

[17] Eisenhauer EA Therasse P Bogaerts J. New response evaluation criteria in solid tumours: revised RECIST guideline (version 1.1). Eur J Cancer. 2009;45:228–247. 19097774 10.1016/j.ejca.2008.10.026

[18] European Association of Urology. EAU - EANM - ESTRO - ESUR - ISUP - SIOG guidelines on prostate cancer. Uroweb.org website. https://uroweb.org/guideline/prostate-cancer/. Accessed March 7, 2023.

[19] Sweat SD Pacelli A Murphy GP Bostwick DG. Prostate-specific membrane antigen expression is greatest in prostate adenocarcinoma and lymph node metastases. Urology. 1998;52:637–640. 9763084 10.1016/s0090-4295(98)00278-7

[20] Seitz AK Rauscher I Haller B. Preliminary results on response assessment using ^68^Ga-HBED-CC-PSMA PET/CT in patients with metastatic prostate cancer undergoing docetaxel chemotherapy. Eur J Nucl Med Mol Imaging. 2018;45:602–612. 29185010 10.1007/s00259-017-3887-x

[21] Grubmüller B Rasul S Baltzer P. Response assessment using [^68^Ga]Ga-PSMA ligand PET in patients undergoing systemic therapy for metastatic castration-resistant prostate cancer. Prostate. 2020;80:74–82. 31614001 10.1002/pros.23919

[22] Aggarwal R Wei X Kim W. Heterogeneous flare in prostate-specific membrane antigen positron emission tomography tracer uptake with initiation of androgen pathway blockade in metastatic prostate cancer. Eur Urol Oncol. 2018;1:78–82. 31100231 10.1016/j.euo.2018.03.010

[23] Rosar F Dewes S Ries M. New insights in the paradigm of upregulation of tumoral PSMA expression by androgen receptor blockade: enzalutamide induces PSMA upregulation in castration-resistant prostate cancer even in patients having previously progressed on enzalutamide. Eur J Nucl Med Mol Imaging. 2020;47:687–694. 31901103 10.1007/s00259-019-04674-0

[24] Plouznikoff N Artigas C Sideris S. Evaluation of PSMA expression changes on PET/CT before and after initiation of novel antiandrogen drugs (enzalutamide or abiraterone) in metastatic castration-resistant prostate cancer patients. Ann Nucl Med. 2019;33:945–954. 31587172 10.1007/s12149-019-01404-2

[25] Gafita A Calais J Grogan TR. Nomograms to predict outcomes after ^177^Lu-PSMA therapy in men with metastatic castration-resistant prostate cancer: an international, multicentre, retrospective study. Lancet Oncol. 2021;22:1115–1125. 34246328 10.1016/S1470-2045(21)00274-6

[26] Scher HI Morris MJ Stadler WM. Trial design and objectives for castration-resistant prostate cancer: updated recommendations from the Prostate Cancer Clinical Trials Working Group 3. J Clin Oncol. 2016;34:1402–1418. 26903579 10.1200/JCO.2015.64.2702 PMC4872347

[27] Vaz S Hadaschik B Gabriel M Herrmann K Eiber M Costa D. Influence of androgen deprivation therapy on PSMA expression and PSMA-ligand PET imaging of prostate cancer patients. Eur J Nucl Med Mol Imaging. 2020;47:9–15. 31654093 10.1007/s00259-019-04529-8

[28] Madan RA Mena E Lindenberg L Choyke PL. With new technology comes great responsibility: prostate-specific membrane antigen imaging in recurrent prostate cancer. J Clin Oncol. 2022;40:3015–3019. 35658513 10.1200/JCO.22.00493 PMC9851688

